# EFFECT OF SECOND TOE-TO-HAND TRANSFER ON THE PLANTAR PRESSURE DISTRIBUTION OF THE DONOR FOOT

**DOI:** 10.1590/1413-785220162401140540

**Published:** 2016

**Authors:** Bing Li, Da-wei Chen, Yun-feng Yang, Guang-rong Yu

**Affiliations:** 1. Tongji University School of Medicine, Shanghai, China. Tongji Hospital, Department of Orthopaedics, Shanghai, China.; 2. Fudan University, Shanghai Medical College, Shanghai Pudong Hospital, Department of Orthopaedics, Shanghai, China.

**Keywords:** Foot, Toes/surgery, Toes/transplantation

## Abstract

**Objective:**

To investigate the effect of second toe-to-hand transfer on the plantar pressure distribution of the donor foot.

**Methods:**

Twelve normal fresh-frozen cadaveric foot specimens were subjected to an axial load of 600 N. An F-Scan plantar pressure analysis system was used to measure the forefoot plantar pressure. The testing was performed under the conditions of intact second toe, second toe removal with the second metatarsal head reserved, and second toe removal in combination with the distal one-third of the second metatarsal, respectively.

**Results:**

The peak pressure of the second metatarsal head was greater than other four forefoot plantar regions. There was no statistically significant change in the forefoot plantar pressure distribution after the second toe was removed (p > 0.05). When the second toe and the distal one-third of the second metatarsal were removed, the forefoot plantar pressure distribution changed significantly (p < 0.05).

**Conclusions:**

An intact second metatarsal is essential for the normal distribution of plantar pressure. Removal of the second toe with the second metatarsal head reserved had little influence on the plantar pressure distribution of the donor foot. Removal of the second toe and distal one-third of the second metatarsal resulted in abnormal plantar pressure distribution.*** Level of Evidence II, Experimental Study.***

## INTRODUCTION

With the development of microsurgery, toe-to-hand transfer has been widely used to reconstruct the fingers of an injured hand. This technique could significantly improve the function of the injured hand. However, reports about the influence of such operation on the function of donor foot are rare. Barca et al.^1^ reported that second toe transfer influenced the function of a donor foot to a lesser extent compared with great toe transfer and was, therefore, preferred. In clinical practice, second toe transfer is most commonly used to reconstruct the thumb. Based on the degree of thumb defect, the second toe transfer with or without the second metatarsal head reserved could be chosen.^2^

The tarsal and metatarsal bones form the foot arch. The metatarsals play an important role in supporting the foot arch, stress transfer, and weight buffering. Currently, a few clinical studies indicate that the second toe transplantation could cause forefoot pain, plantar callus and forefoot deformity.^3^ ^-^ ^5^ However, there is no report of relevant biomechanical research. We hypothesized that the second toe removal with and without the second metatarsal head reserved would produce different biomechanical effects on the donor foot. The purpose of the current study was to determine the effect of removal of the second toe at different levels on the plantar pressure distribution of the donor foot.

## METHODS

This study complied with the Helsinki Declaration regarding research on human subjects. Ethical approval was obtained from the Human Research Ethics Committee, Tongji Hospital, Tongji University School of Medicine, Shanghai, China (KYSB-2014-18). The specimen donors or the next of kin agreed that the specimens were used in medical research, and signed a Free and Informed Consent Term.

Twelve normal fresh-frozen cadaveric foot specimens from twelve donors were examined. The average age of the donors at death was 57.4 years old (range, 45-71 years old). The specimens were amputated 10 cm below the knee joint. Evident preexisting foot abnormalities were excluded by visual inspection and review of the medical history. X-rays were performed to exclude osteoarthritis, previous fractures, tumors, osteonecrosis, and foot deformities. Before the experiments the specimens were stored in a refrigerator at a temperature of -20°C.

The specimens were removed from the refrigerator and unfrozen naturally at room temperature 24h before the experiment. The skin, muscles and other soft tissues were removed 10cm above the ankle until the tibia and fibula were exposed. Skin and ligaments around the ankle joint were kept intact. Part of the proximal fibula was removed to make it about 5cm shorter than the tibial stump, in order to facilitate loading and fixation.

**Figure 1:**
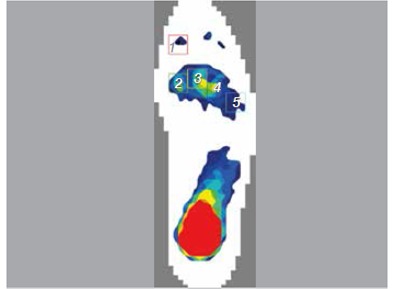
Representative image showing plantar pressure distribution areas: region 1, hallux; region 2, first metatarsal head; region 3, second metatarsal head; region 4, third to fourth metatarsal head; region 5, fifth metatarsal head (red, yellow, green and blue indicate plantar pressure intensity from high to low).

The specimen was placed on a loading platform. The proximal tibia was fixed on top of the loading machine (CSS-44010, Crims Co. Ltd, Changchun, China). The specimen was placed with the ankle in neutral position, keeping the tibia perpendicular to the loading platform. A plantar pressure analysis system F-scan (Tekscan Inc., Boston, MA, USA) was used to measure plantar pressure during loading. An insole sensor film was placed between the plantar foot and the loading platform. The specimen was subjected to a 600N axial load, which was equivalent to the body weight of a 60kg individual. The F-Scan system was calibrated under 600 N axial load. Data collection period was adjusted to 8sec, with a collection frequency of 50 frames/sec. The software provided with the F-Scan system was used to record the plantar pressure. Similar to the study by Yu et al.,^6^ five boxes inserted into the plantar pressure distribution areas were used to collect the peak plantar pressure of the hallux, first metatarsal head, second metatarsal head, third to fourth metatarsal head, and fifth metatarsal head. (Figure 1) Measurements were carried out in the following three situations: (1) Intact second toe; (2) Removal of the second toe with the second metatarsal head reserved; and (3) Removal of the second toe and distal third of the second metatarsal.

Statistical analysis was made using SPSS version 17.0 for Windows (SPSS Inc., Chicago, USA). One-way analysis of variance (ANOVA) was used to test for significant differences in plantar pressure across the groups. Where differences existed between groups, the Tukey's post-hoc test was used to make multiple comparisons.^7^ The level of significance was set at p < 0.05.

## RESULTS

There were significant differences in the peak pressures of the five forefoot plantar regions when the second toe was kept intact (p < 0.05). The peak pressure of the second metatarsal head was greater than the other four forefoot plantar regions

(p < 0.05). Under a 600N axial load, the peak pressure of the five forefoot plantar regions ranked in the following descending order: the second metatarsal head, third to fourth metatarsal head, first metatarsal head, fifth metatarsal head, and hallux. (Table 1) 

**Table 1:** Summary of data on the peak plantar pressure for the specimens with intact second toe, second toe removal, and part second metatarsal removal.

Region	Peak pressure (KPa)	
	Intact (n = 12)	Second toe removal (n = 12)	Part second metatarsal removal (n = 12)
Hallux	14.66 ± 3.11	17.25 ± 3.25	22.70 ± 3.03*
MT1	35.04 ± 4.42	39.19 ± 4.24	68.26 ± 6.26*
MT2	66.17 ± 6.05	70.25 ± 6.48	/
MT3-4	52.68 ± 5.07	56.90 ± 5.13	106.44 ± 7.19*
MT5	22.02 ± 3.50	24.95 ± 3.08	35.95 ± 4.07*

Data presented as mean ± Standard deviation (SD); *indicates a significant difference between intact and part second metatarsal removal groups (p < 0.05); MT: metatarsal head.

**Figure 2:**
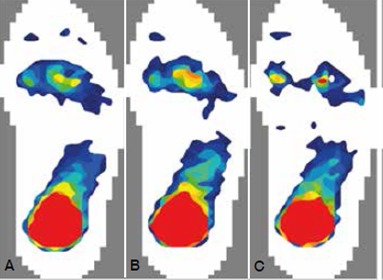
Plantar pressure distribution under three different conditions: (A) Intact second toe. (B) Second toe removal. (C) Second toe removal in combination with the distal one-third of the second metatarsal (red, yellow, green and blue indicate plantar pressure intensity from a high to low).

There was no statistically significant change in the peak pressure of all five forefoot plantar regions after the second toe was removed with the second metatarsal head reserved (p > 0.05). However, when the second toe together with the distal one-third of the second metatarsal was removed, the forefoot plantar pressure distribution changed significantly (p < 0.05). (Table 1, Figure 2)

## DISCUSSION

The second metatarsal is an important part of the foot arch. It takes a part in weight bearing and keeps the stability of the foot arch. In the anatomical structures of the foot, the three cuneiform bones are embedded into each other to form a convex-upward arch structure at the tarsometatarsal joint.^8^ ^,^ ^9^ The medial and lateral cuneiforms distally protrude beyond the intermediate cuneiform forming, thus, a cavity to accommodate the base of the second metatarsal. This cavity allows the second metatarsal to firmly embed into it. Such structural characteristic limits the activity of the second metatarsal. As a result, the second metatarsal plays a major role in resisting bending and shearing forces, which are mainly transferred through the second metatarsal during gait.^10^ ^-^ ^12^

The second and third metatarsals serve as the middle column of the foot. The range of motion of these two bones at the tarsometatarsal joint is very small. In contrast, the first tarsometatarsal joint, as the medial column and the fourth and fifth tarsometatarsal joints and the lateral columns show a relative large range of motion.^13^ ^-^ ^15^ When the foot bears a weight, the medial and lateral columns appropriately buffer the stress, while the second and third metatarsals give strong support. Therefore, the stress borne by the second and third metatarsals is relative larger. Hennig and Milani^16^ found that whether under static or dynamic conditions, the peak pressure beneath the second and third metatarsals were much higher than that beneath the first and fifth metatarsal heads. Our findings agree with this point. 

Our results showed that the peak pressure beneath the second metatarsal head was the largest among the five plantar regions. This indicates that, under static weight bearing, the second metatarsal supports the largest stress. To some extent, this may explain why the second metatarsal is prone to stress fracture.^12^ ^,^ ^17^ Our results were comparable to the reports by other authors. Kanatli et al.^18^ tested the forefoot plantar pressure in 16 normal subjects, and found that the plantar region with the largest average pressure during standing was under the second and third metatarsal head (7.96 N/cm^2)^. The pressure of the first metatarsal head was 4.86 N/cm^2^, and the fourth to fifth metatarsal head was 6.26N/cm^2^. Hinz et al.^19^ measured the plantar pressure of 26 soldiers. Their results indicated that during walk the forefoot pressure ranked in the following descending order: second metatarsal head, third metatarsal head, first metatarsal head, fourth metatarsal head and fifth metatarsal head, which is similar to our results.

Our results showed that the peak pressure beneath the second metatarsal head was the largest among the five plantar regions. This indicates that, under static weight bearing, the second metatarsal supports the largest stress. To some extent, this may explain why the second metatarsal is prone to stress fracture.^12^ ^,^ ^17^ Our results were comparable to the reports by other authors. Kanatli et al.^18^ tested the forefoot plantar pressure in 16 normal subjects, and found that the plantar region with the largest average pressure during standing was under the second and third metatarsal head (7.96 N/cm2). The pressure of the first metatarsal head was 4.86 N/cm^2^, and the fourth to fifth metatarsal head was 6.26N/cm^2^. Hinz et al.^19^ measured the plantar pressure in 26 soldiers. Their results indicated that during gait the forefoot pressure ranked in the following descending order: second metatarsal head, third metatarsal head, first metatarsal head, fourth metatarsal head and fifth metatarsal head, which is similar to our results. Gu et al.^3^ reported 212 cases of second toe-to-hand transfer, of which 171 cases had the second metatarsal head reserved and 41 cases had part of the second metatarsal resected. The patients with the second metatarsal head preserved had a much lower incidence of donor foot pain, running restriction, plantar callus, and forefoot deformity. Lui et al.^5^ reported one case of second toe-to-hand transfer with part of the second metatarsal removed. In 30 year follow-up, the patient experienced persistent forefoot pain at the third metatarsophalangeal joint and fifth metatarsal head. Meanwhile, the patient had hallux valgus, crossover third toe and medial deviation of the fourth and fifth toes. As a result, the second metatarsal reconstruction had to be performed to relieve pain and improve the foot function. These reports indicated that, in comparison to resection of part of the second metatarsal, the second toe transfer with the second metatarsal head reserved would not greatly influence the function of the donor foot. 

To the best of our knowledge, this study is the first report on the effect of second toe transfer on the plantar pressure distribution of the donor foot. Our tests showed that after the second toe was removed with the second metatarsal head reserved, the peak pressure of each plantar region of the forefoot did not increase significantly. This indicates that second toe removal with the second metatarsal head reserved might have little impact on the weight bearing function of the foot in the short term. After the second toe was removed in combination with the distal one-third of the second metatarsal, the plantar pressure of the second metatarsal head disappeared and the plantar pressure of other four plantar regions increased significantly. The load was mainly transferred to the first, third, and fourth metatarsals. The peak pressure beneath the first metatarsal head increased 97%, and of the third to fourth metatarsal head increased 103%. Due to the second metatarsal defect, the plantar pressure distribution became abnormal. We consider that the abnormal distribution of the plantar pressure might further lead to forefoot deformity, foot arch collapse, fatigue fractures, and a series of other complications of the donor foot in the long term.

There were some limitations to our study. Firstly, the number of the specimens was too small. Second, only static axial load was applied. Dynamic loading to simulate the pressures exerted during walking could not be performed in our equipment. Third, only a load of 600N was applied in the testing. A much heavier load or repeated loading might produce different results. Furthermore, the cadaveric study could only reflect the acute change of the parameters, and a clinical comparative study ought to be performed to see the long-term effects.

## CONCLUSION

An intact second metatarsal took an important part in the normal distribution of plantar pressure. Removal of the second toe with the second metatarsal head reserved had little influence on the plantar pressure distribution of the donor foot. Removal of the second toe and distal-third of the second metatarsal resulted in abnormal plantar pressure distribution. In the second toe-to-hand transfer, the second metatarsal head should be reserved as much as possible to reduce the complications on the donor foot.
